# Trajet de soins des personnes âgées à Bobo-Dioulasso, au Burkina Faso: une enquête transversale

**DOI:** 10.11604/pamj.2015.20.128.5822

**Published:** 2015-02-13

**Authors:** Hervé Hien, Abdramane Berthé, Blahima Konaté, Maxime koiné Drabo, Fatoumata Tou, Désiré Somda, Fatoumata Badini-Kinda, Jean Macq

**Affiliations:** 1 Centre MURAZ, Bobo-Dioulasso, Burkina Faso; 2 Institut de Recherche en Sciences de la Santé, Bobo-Dioulasso, Burkina Faso; 3 Laboratoire National de santé Publique, Ouagadougou, Burkina Faso; 4 Université de Ouagadougou UFR/SH, Burkina Faso; 5 Université Catholique de Louvain, IRSS, Bruxelles, Belgique

**Keywords:** Trajet, personnes âgées, soins formels, soins informels, journey, elderly, formal care, informal care

## Abstract

**Introduction:**

En Afrique, il y n'a pas encore une maitrise du trajet de soins des personnes âgées pour construire des modèles de soins adaptés afin d'améliorer leur prise en charge. L'objectif de cette étude était de décrire le trajet de soins des personnes âgées à Bobo-Dioulasso.

**Méthodes:**

Nous avons réalisé une étude transversale à prédominance qualitative avec des personnes âgées vivant dans les ménages dans la ville de Bobo-Dioulasso de septembre à novembre 2012. Nous avons sélectionné 30 personnes âgées de manière raisonnée dans 22 secteurs. Des entretiens qualitatifs ont été réalisés. Le recours aux soins formels, informels et mixtes a été analysé.

**Résultats:**

Le trajet de soins des personnes âgées à Bobo-Dioulasso était à prédominance mixte: elles utilisaient à la fois pour le même épisode de maladie les services publics et privés l'automédication à domicile, la médecine traditionnelle, l'utilisation des médicaments du marche informel de la rue. Les premiers recours aux soins étaient à l'initiative des personnes âgées elles-mêmes. Les recours aux soins formels étaient largement utilisés par les personnes âgées qui avaient un revenu de pension.

**Conclusion:**

Devant la complexité de la prise en charge des personnes âgées présentant plusieurs pathologies et ayant différents recours des soins il y a une nécessité d'orienter le système d'offre de soins vers une coordination dite « collective ».

## Introduction

Les personnes âgées sont de plus en plus reconnues dans les pays en développement comme une population prioritaire [1–3] Elles constituent en effet une population vulnérable [4]. Elles ont encore des besoins nutritionnels, matériels et financiers non couverts [5]. Pour la prise en charge de leurs maladies chroniques, ces personnes âgées sont en contact avec de multiples acteurs du système de soins. Sur le plan médical, elles bénéficient d'une succession d'examens diagnostiques, de traitements médicaux ou chirurgicaux, et leur état de santé suit un cours variable: stabilité, aggravation, amélioration. Cette variation de leur état santé nécessite des recours multiples de soins. La maîtrise des trajectoires de soins par le patient et ses soignants est importante: elle est un gage de qualité des soins, de la qualité de vie du patient et d'efficience médico-économique pour le système de soins [6]. Pourtant en Afrique et au Burkina Faso, il y n'a pas encore une maitrise des trajectoires de soins des personnes âgées malades pour construire des modèles de soins adaptés au contexte local pour la prise en charge des pathologies chroniques. La littérature scientifique dans les pays en développement et en particulier en Afrique est pauvre sur la question. Les quelques résultats publiés sont également divergents sur le type de recours le plus utilisé par les personnes âgées. Trois types de recours de soins ont été identifiés dans la littérature africaine pour les personnes âgées: les soins formels, informels et mixtes. Selon Peter C en 2008 puis Berthé A en 2012, les PA reçoivent des soins informels de la part d'elles mêmes, des membres de la famille ou d'amis à domicile [4, 7]. Cependant une prévision d'un recul de l'offre de soins informels est annoncée [7]. Les raisons évoquées sont la diminution du nombre des personnes âgées vivant avec leurs enfants, la hausse du nombre d'ainés vivant seuls, la baisse du nombre de femmes à dispenser des soins au fur et à mesure que leur taux d'emploi augmente.

Selon Ndeindo N en 2012, débuter un parcours de soins en recourant à un service de santé n'empêchait pas de consulter ensuite un guérisseur ou d'acheter des médicaments sur le marché informel [8]. Selon Mveing S en 2008, une personne âgée malade pouvait utiliser plusieurs secteurs de consultation: le secteur formel (public ou parapublic, privé laïc, privé confessionnel) et le secteur informel (vendeur informel de médicaments, tradipraticiens, etc.). Cependant, l'accessibilité financière guide plus les personnes âgées à recourir aux soins officiels [9]. Ces différentes trajectoires de soins utilisées par les personnes âgées témoignent de la complexité (pas de couverture maladie, faible accessibilité financière, géographique et culturelle des soins) du contexte de soins en Afrique. En effet la faible accessibilité financière des soins par les personnes âgées, couplée à la perception négative de la vieillesse, au vécu des maladies et des soins par les personnes âgées et la place des réseaux sociaux [9] ont été identifiés pour expliquer cette multiplicité des recours de soins. Des interventions isolées ont été proposées pour améliorer la prise en charge des personnes âgées. La plupart de ces interventions n'empruntent pas la perspective systémique en santé publique. Elles sont toujours pour la plupart focalisées sur le secteur public témoignant encore de la maitrise insuffisante des trajectoires de soins pour la prise en charge des personnes âgées malades en Afrique. L'objectif de cette étude était de décrire le trajet de soins des personnes âgées à Bobo-Dioulasso.

## Méthodes

### Type d’étude

Nous avons réalisé une étude transversale à prédominance qualitative avec les personnes âgées vivant dans les ménages dans la ville de Bobo-Dioulasso en 2012.

### Cadre de l’étude

Le système sanitaire du Burkina Faso comprend les sous-systèmes suivants: le sous- système public de santé, le sous-système privé de santé et le sous-système de la médecine et de la pharmacopée traditionnelle. Les structures publiques de soins sont organisées en trois niveaux: le premier niveau est constitué des services de première ligne (les centres de santé et de promotion sociale (CSPS), les centres médicaux (CM), les dispensaires et autres maternités isolées) et les hôpitaux de première référence. Le deuxième niveau comprend les centres hospitaliers régionaux (CHR) et enfin le troisième niveau regroupe les trois hôpitaux universitaires ou CHU du pays. La gestion du système public de soins se fait à trois niveaux: le niveau central, organisé autour du cabinet du Ministre de la santé et du Secrétariat général, est chargé de l’élaboration des politiques, de la mobilisation des ressources, du contrôle de gestion et de l’évaluation des performances, le niveau intermédiaire comporte 13 directions régionales de la santé chargées de la coordination et de l'appui aux districts, le niveau périphérique compte actuellement 63 districts sanitaires dont les équipes cadres gèrent les services de première ligne et de première référence. L’étude a été réalisée dans la ville de Bobo-Dioulasso qui est la deuxième ville du Burkina Faso. L'offre de soins au premier niveau est organisée par deux districts sanitaires comptant 36 CSPS (27 pour le district sanitaire de Dafra et 9 pour le district sanitaire de Dô), 6 maternités ou dispensaires et 2 hôpitaux. Des structures de santé privées et confessionnelles participent au système d'offre de soins. Le dernier niveau de référence est représenté par un CHU. Il n'y pas de niveau intermédiaire de soins à Bobo-Dioulasso. L'offre de soins informels est assurée par les tradipraticiens, les vendeurs ambulants de médicaments [10].

### Population d’étude et période de l’étude

L'enquête a été réalisée de septembre à novembre en 2012 à Bobo-Dioulasso. La population d’étude était constituée des personnes âgées vivant à Bobo-Dioulasso pendant la période d’étude. Les critères d'inclusion étaient:1) Être une personne âgée de 60 ans et plus, 2) vivant dans la ville de Bobo-Dioulasso, 3) ayant donné son consentement pour participer à l’étude

### Echantillonnage et taille de l’échantillon

Nous avons sélectionné de manière raisonnée 30 personnes âgées vivant dans les ménages dans chacun des 22 secteurs de la ville de Bobo-Dioulasso. Après avoir administré un questionnaire à un échantillon représentatif de personnes âgées de Bobo-Dioulasso (volet quantitatif de notre étude pour documenter les pathologies chroniques), nous avons sélectionné celles qui ont été malades au cours des deux dernières semaines précédent la collecte des données. A partir de cette liste, nous avons choisi les personnes âgées par secteur géographique en fonction de leur taille pour repartir documenter qualitativement leur trajet de soin. Toutes les personnes sollicitées ont participé à l’étude.

### Collecte des données

#### Définition des variables

Trajet de soins: se réfère aux déplacements d'un patient dans le système de soins (recours aux soins) [6] au cours d'un même épisode de maladie (du début de la maladie jusqu’à la guérison totale ou à la maîtrise ou contrôle de la maladie.)

Les soins formels: ce sont des soins délivrés dans un service de santé par des prestataires des soins formés [7, 8]. Dans notre étude les soins formels concernaient ceux administrés dans les services de santé publics et privés.

Les soins informels: les soins informels sont des soins non dispensés dans un service de santé. Ces soins concernent la médecine traditionnelle, l'automédication, l'utilisation des médicaments du marché, les services l'entourage familial [7, 8].

Les soins mixtes: c'est l'utilisation à la fois des deux types des soins informels et formels au cours du même épisode de maladie [8].

#### Techniques et outils de collecte des données

Des entretiens individuels ont été réalisés avec les personnes âgées. Quand la personne âgée n’était pas capable de répondre aux questions, un aidant proche l'assistait. Un questionnaire semi structuré a été utilisé pour la collecte des données. Les principaux items du questionnaire étaient: 1) les caractéristiques sociodémographiques, 2) l’épisode de maladie, 3) les différents recours de soins utilisés par la personne âgée lors de l’épisode de maladie, 4) le soutien de la famille pour le recours de soins et la gestion de la maladie et des médicaments, 5) les raisons de choix des différents recours.

#### Mode de collecte des données

La collecte des données a été réalisée par binôme (deux enquêteurs) médecins et sociologue. Cette étude a été réalisée au décours d'une enquête ménage dans la ville pour identifier les problèmes de santé des personnes âgées vivant à domicile. Pour cette étude, le médecin avait pour rôle de collecter les épisodes de maladies. Le sociologue procédait aux entretiens. Une formation des enquêteurs sur l'utilisation du questionnaire, des techniques de collecte et de repérage des ménages a été réalisée. Un pré-test du questionnaire a été réalisé avant la collecte des données.

### Analyse des données

L'analyse des données a été faite à 2 niveaux: (1) à description de l’échantillon (âge, sexe, scolarité, vivre en couple); (2) les indicateurs évalués: les caractéristiques du premier recours: primaires (à l'initiative de la personne âgée ou de la famille) ou secondaire (à l'initiative d'un personnel de santé), la description des différents recours de soins formels, informels ou mixtes, les raisons de choix des différents recours.

### Considérations éthiques

Le protocole de recherche a reçu l'accord du comité d’éthique pour la recherche en santé du Burkina Faso. Une notice d'information et un formulaire de consentement ont été utilisés pour recueillir le consentement des participants à l’étude. L'anonymat et la confidentialité des données ont été assurés tout au long de l'enquête: collecte, saisie et analyse des données.

## Résultats

### Description de l’échantillon

Nous avons interrogé 30 personnes âgées au cours de notre étude. L’âge médian était de 65 ans (IQ: 62; 71). Le sexe ratio H/F était 18/12. Les personnes âgées mariées vivant avec leur conjoint(e) représentaient 70% (21/30). Le niveau d'instruction était respectivement 62,1% (18/29) pour les scolarisés (école coranique, primaire, secondaire et universitaire) et 37,9% (11/29) pour les non scolarisés. Les personnes âgées qui avaient un revenu régulier bénéficiant d'une pension de retraite représentaient 44,8% (13/29).

### Caractéristiques du premier recours des soins

**Figure 1:**
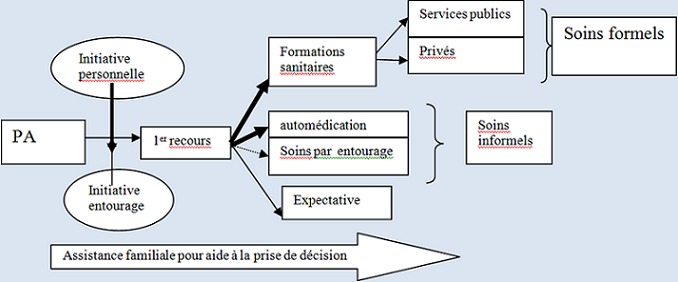
Caractéristiques du premier recours aux soins chez les personnes âgées à Bobo-Dioulasso, 2012

Les principaux épisodes de maladie qui ont conduit aux recours de soins étaient les troubles neurologiques 40% (12/30) et les troubles digestifs 20% (6/30) Tableau 1. Le premier recours aux soins à l'initiative des personnes âgées était de 84% (21/25). Le recours aux soins à l'initiative d'autres personnes était de 16% (4/25): il se faisait soit par un personnel infirmier à partir du domicile de la personne âgée soit par un membre de la famille. Le premier recours aux soins pour les épisodes de maladie était représenté par une consultation dans une formation sanitaire publique ou privée 36,7% (11/30), une automédication 26,7% (8/30), une abstention ou expectative 26,7% (8/30), une consultation chez un tradithérapeute 10% (3/30). Les 8 personnes qui s’étaient abstenues de recevoir des soins ont déclaré avoir pris cette initiative dans le but de supporter la maladie. Ces personnes âgées étaient quasi représentées par des hommes (5/6) et vivaient tous en couple. L'automédication a été pratiquée pour les médicaments tels que l'aspirine, le paracétamol et des médicaments traditionnels (poudre et décoctions de plantes). Lors du premier recours thérapeutique, les formations sanitaires visitées pour « des soins formels » étaient les centres de santé de premier niveau publique, le second niveau (CHU) et les structures privées de la ville (4/11). Pendant cette démarche de soins, les membres de l'entourage qui ont assisté la personne âgée étaient représentés 1) 40% (12/30) par des descendants (les belles filles, les fils, filles et les petits enfants), 2) 26,7% (8/30) par une personne du voisinage du domicile 3) 33,3% (10/30) par des collatéraux (les frères, les époux (ses). L'assistance de l'entourage était caractérisée par un soutien moral ou financier, de conseils sur les médicaments ou de don de médicaments ou pour inciter la personne âgée à entreprendre la démarche de soins ou la consommation de médicaments. Cet entourage a participé à l'aide à la prise de décision pour le premier recours aux soins. La Figure 1 présente les caractéristiques du premier recours thérapeutique.

**Tableau 1:** Épisodes de maladie des personnes âgées

Episodes de maladies	Nombre de personnes concernées
Troubles neurologiques (céphalées, perte de connaissance)	12/30
Troubles digestifs (douleur abdominale, vomissements)	6/30
Troubles ostéo-articulaires (chute, douleur articulaire diffuse)	3/30
Troubles respiratoires (toux, douleur thoracique)	2/30
Troubles cardiovasculaires (palpitations, vertiges)	2/30
Troubles urinaires (polyurie, dysurie)	2/30
Troubles métaboliques (œdèmes des chevilles)	1/30
Troubles oculaires (prurit oculaire, œil rouge)	1/30
Troubles stomatologiques (douleur dentaire)	1/30

### Trajet de soins

**Figure 2:**
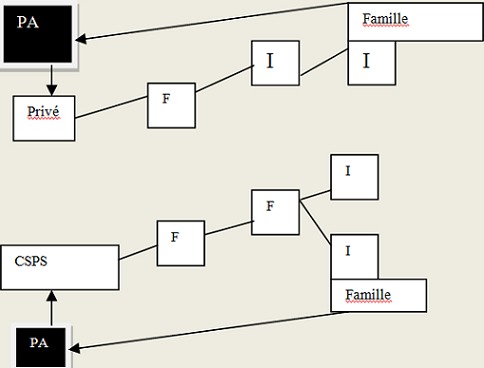
Trajet de soins dont les premiers soins étaient formels

**Figure 3:**
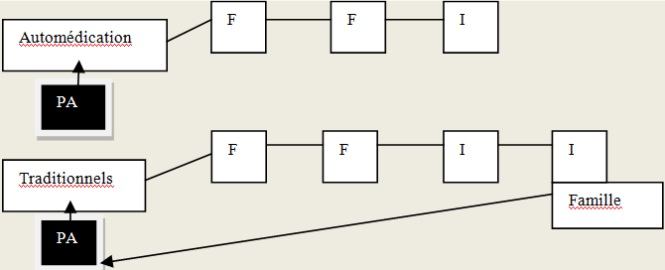
Trajet de soins dont les premiers soins étaient informels

**Figure 4:**
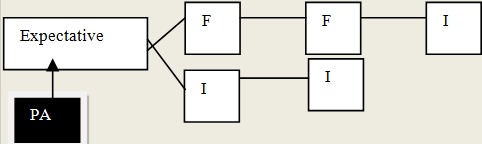
Trajet de soins dont les premiers soins étaient l'expectative (à domicile)

**Figure 5:**
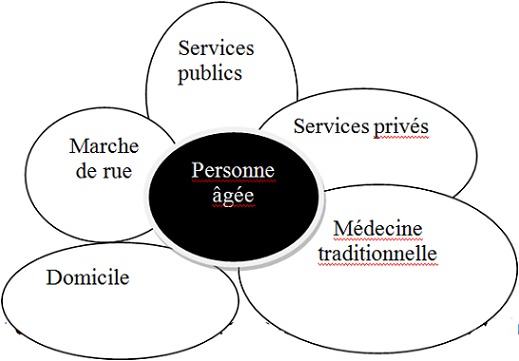
Composantes des recours aux soins du sujet âgé à Bobo-Dioulasso

Trois types de recours aux soins ont été identifiés: un recours aux soins formels, soins informels et aux soins mixtes. En dehors de ceux qui ont eu comme premiers recours aux soins le CHU soit 10% (3/30), la plupart, soit 90% des personnes âgées ont eu un parcours mixte de soins: 37% (11/30) pour les recours aux soins formels et 53%(16/30) pour les soins informels. Pour le trajet dont les premiers recours de soins étaient formels, les personnes âgées étaient la plupart celles qui avaient un revenu 81,8% (9/11). Le plus long parcours était celui dont le premier recours de soins était des soins informels dits « traditionnels ». Dans ce cas, les personnes âgées ont parcouru 4 étapes pour recevoir des soins (des soins traditionnels, puis dans un cabinet privé, ensuite des soins traditionnels et enfin des soins par un membre de la famille. Dans tous cas de recours aux soins informels et formels, la famille étaient pour la plupart de temps impliquée. Elle était présente dès le début de l’épisode de la maladie, du premier recours et à la fin du parcours de soins soit pour donner des soins et des médicaments à la personne âgée à domicile. Le recours aux soins dont les premiers soins étaient l'expectative a été notée dans notre étude. Les Figure 2, Figure 3, Figure 4 présentent les différents recours aux soins des personnes âgées selon que les premiers soins étaient formels, informels ou l'expectative. La Figure 5 présente les composantes nécessaires des recours aux soins du patient au sein d'un système d'offre de soins dans le contexte local de Bobo-Dioulasso

### Motifs des recours aux soins

#### La faible accessibilité géographique ou financière

Elle a été notée par les personnes âgées pour expliquer le recours vers les soins informels (automédication, soins traditionnels). Les personnes âgées ont déclaré s’être rendues dans les centres de santé qui étaient plus proches de leur domicile et particulièrement les cliniques privés. La famille a été d'une aide quand les frais de consultation ont été déclarés non abordables par la personne âgée. (2000 à 12 000 FCFA).

#### La qualité de l'interaction entre la personne âgée, le personnel de soins et la famille

Pendant les trajets de soins formels, les personnes âgées ont déclaré avoir eu recours à du personnel paramédical et du personnel médical. Selon les personnes âgées, plusieurs contacts précédents ont eu lieu avec ce personnel de soins à qui elles avaient confiance. Dans la plupart des cas, il y avait soit des liens de parenté avec la famille (cousin de la belle fille, neveu ami de la maison) ou de voisinage (voisin de la maison ou de quartier) entre les personnes âgées et les soignants.

#### La mobilisation familiale

La forte mobilisation de la famille autour de la personne âgée pendant l’épisode de santé a permis le soutien financier, moral, émotionnel, informationnel, le soutien dans la mobilité (le transport des personnes âgées vers les thérapeutes et les structures de santé), dans les activités de la vie quotidienne ou domestique.

#### Des croyances et les expériences des personnels âgées avec les soins

L'expérience vécue avec certaines structures de soins a été notée par les personnes âgées. Les soins traditionnels ont été considérés comme habituels pour expliquer ce type de recours. La connaissance et la confiance portées aux professionnels de santé et des tradithérapeutes ont été également notées par les personnes âgées.

## Discussion

Le trajet de soins des personnes âgées à Bobo-Dioulasso était à prédominance mixte (90% des personnes âgées). Les personnes âgées utilisaient à la fois les recours aux soins formels (services publics et privés) et informels (automédication à domicile, médecine traditionnelle, utilisation des médicaments du marché informel) pour leurs soins. Les premiers recours aux soins étaient à l'initiative des personnes âgées elles-mêmes. Les recours aux soins formels étaient largement (81,8% (9/11) utilisés par les personnes âgées qui avaient un revenu de pension. Le trajet de soins le plus long commençait par les soins informels traditionnels. L'entourage familial était présent depuis l'initiative à la démarche de soins qu'elle soit formelle ou informelle pour aider à la prise de décision et à l'accompagnement de la prise en charge. En Afrique en général, il est reconnu que lorsqu'une personne âgée est malade, elle peut utiliser plusieurs recours de soins [9]. Nous avons trouvé dans notre étude que le trajet de soins de personnes âgées était complexe dynamique donc non figé ou unilinéaire. Le coût (accessibilité financière), la distance (en cas de nécessité de plusieurs contacts pour les soins liés au même épisode), la satisfaction des interactions ou l'amélioration de son état (accessibilité culturelle) expliquent cet aspect dynamique et complexe de ces trajets de soins de soins. Les personnes âgées utilisaient à la fois les recours formels de soins et les recours informels. Le recours informel aux soins était varié (médecine traditionnelle, médicaments du marché local et de la famille). Tous les secteurs de santé sont utilisés pour les soins des pathologies des personnes âgées. Dans ces trajets de soins, la place de l'automédication, l'achat des médicaments sur le marché de rue, la médecine traditionnelle est importante soit 60% (18/30) des personnes âgées. Tous ces soins informels soutenus par la famille s'administrent finalement à domicile. Durant tous les recours de soins des personnes âgées, la famille était présente. La famille constitue une provision importante de recours de soins à préserver. Pourtant, l'OMS laisse penser des inquiétudes sur la pérennité des ce recours pour la prise en charge des personnes âgées. Les raisons évoquées sont la diminution du nombre des personnes âgées vivant avec leurs enfants, la hausse du nombre d'ainés vivant seuls, la baisse du nombre de femmes à dispenser des soins au fur et à mesure que leur taux d'emploi augmente [7].

Dans notre étude, les recours aux soins dans les services publics n'empêchent pas les personnes âgées d'utiliser d'autres recours. Tous ces recours engendrent des prises de médicaments de qualité inconnue de soins divers pour des personnes âgées déjà fragilisés par les pathologies chroniques et le vieillissement. Louise S en 2011 souligne que le parcours du patient prend en compte le domicile, l'hôpital; les réseaux des professionnels de soins en ville et au centre le patient [9]. Les principaux motifs qui guidaient vers les différents recours aux soins formels privés étaient l'accessibilité géographique et financière et culturelle. Le plus souvent les personnes âgées ont recouru à des structures de soins (formels et/ou informels) où, elles connaissaient un soignant pour être bien accueillies, bien écoutées/diagnostiquées donc bien soignées. La confiance à ce parent-soignant était plus élevée. L'accessibilité culturelle aux structures de soins est donc déterminante. En général, les patients aiment à se rendent dans les structures de soins où les manières d'agir envers autrui, de penser autrui et/ou sa maladie et la manière d’être des soignants (la culture des soignants) sont relativement plus proches de leur propre culture. C'est ce qui explique souvent le recours aux tradithérapeutes réputés pour leur qualité de communication (échange) avec les malades. Souvent chez ces thérapeutes, c'est l'efficacité symbolique de leurs interactions avec les malades (rites de soins, concordance de la nosographie/étiologie avec celui des malades, médicaments) qui facilite la guérison de ceux-ci.

Les résultats que nous avons trouvés dans notre contexte appelle à réfléchir sur une approche systémique tenant compte de tous les différents recours aux soins utilisés par les personnes âgées pour leur meilleure prise en charge. (Figure 5). Les composants à prendre en compte sont les services publiques, les services privés, la médecine traditionnelle, les soins à domicile, la vente libre des médicaments dans la rue. Dans cette approche, la place des soins à domicile qu'ils soient réalisés par du personne médical ou par un membre de la famille est importante. La prise en charge des personnes âgées avec des pathologies chroniques est complexe. Tous les secteurs et les acteurs de la santé doivent être pris en compte pour développer des stratégies efficaces, pérennes, adaptées aux besoins et aspirations des personnes âgées en Afrique.

## Conclusion

Devant la complexité de la prise en charge des personnes âgées présentant plusieurs pathologies et ayant différents recours des soins il y a une nécessité d'orienter l'offre de soins vers une coordination dite « collective » des soins [11]. Cette approche promeut une pluridisciplinarité. Le rôle d'un coordonnateur de soins est souhaité. Il jouerait le lien avec la famille pour développer la prise en charge des personnes âgées entre le secteur formel et informel. Cette approche permettrait d'augmenter le nombre et la qualité des interactions entre les acteurs de la santé autour de la personne âgée avec des multimorbidités. Cela permettrait ainsi de maîtriser également l'aide apportée par le système de soutien social que représente l'offre de soins informel.
